# Transcriptome profiling of *caspase-2* deficient *EμMyc* and *Th-MYCN* mouse tumors identifies distinct putative roles for caspase-2 in neuronal differentiation and immune signaling

**DOI:** 10.1038/s41419-018-1296-0

**Published:** 2019-01-22

**Authors:** Loretta Dorstyn, Emily Hackett-Jones, Andrej Nikolic, Murray D. Norris, Yoon Lim, John Toubia, Michelle Haber, Sharad Kumar

**Affiliations:** 1 Centre for Cancer Biology, University of South Australia and SA Pathology, GPO Box 2471, Adelaide, SA 5001 Australia; 2 Children’s Cancer Institute Australia for Medical Research, Lowy Cancer Research Centre, UNSW, Sydney, NSW 2052 Australia

## Abstract

Caspase-2 is a highly conserved cysteine protease with roles in apoptosis and tumor suppression. Our recent findings have also demonstrated that the tumor suppression function of caspase-2 is context specific. In particular, while *caspase-2* deficiency augments lymphoma development in the *EμMyc* mouse model, it leads to delayed neuroblastoma development in *Th-MYCN* mice. However, it is unclear how caspase-2 mediates these differential outcomes. Here we utilized RNA sequencing to define the transcriptomic changes caused by *caspase-2* (*Casp2*^*−/−*^) deficiency in tumors from *EμMyc* and *Th-MYCN* mice. We describe key changes in both lymphoma and neuroblastoma-associated genes and identified differential expression of the EGF-like domain-containing gene, *Megf6*, in the two tumor types that may contribute to tumor outcome following loss of *Casp2*. We identified a panel of genes with altered expression in *Th-MYCN/Casp2*^*−/−*^ tumors that are strongly associated with neuroblastoma outcome, with roles in melanogenesis, Wnt and Hippo pathway signaling, that also contribute to neuronal differentiation. In contrast, we found that key changes in gene expression in the *EμMyc/Casp2*^*−/−*^ tumors, are associated with increased immune signaling and T-cell infiltration previously associated with more aggressive lymphoma progression. In addition, Rap1 signaling pathway was uniquely enriched in *Casp2* deficient *EμMyc* tumors. Our findings suggest that *Casp2* deficiency augments immune signaling pathways that may be in turn, enhance lymphomagenesis. Overall, our study has identified new genes and pathways that contribute to the caspase-2 tumor suppressor function and highlight distinct roles for caspase-2 in different tissues.

## Introduction

The role of caspases in tumor suppression has been largely ascribed to their established function in cell death^1^. Recent experimental evidence also suggests that non-apoptotic roles for caspases, including cell proliferation, inflammation, migration, or invasion, can contribute to tumorigenesis^2,3^. These features are well-established hallmarks of cancer, both independently and in co-operation with cell death evasion^4^, and may determine caspase contribution to both tumor suppression and tumor promotion in certain contexts^3^. While the mechanisms that regulate caspase functions in tumorigenesis are still unclear, there is evidence to suggest that caspases mediate their differential functions in a developmental and tissue-specific manner^1,2^.

Caspase-2 is the most evolutionarily conserved member of the mammalian caspase family^5^, with roles in apoptosis^6^, tumor suppression^7–11^, genomic stability^12^, DNA damage response^12,13^, oxidative stress response, metabolism, and ageing^6,14–18^. While mice deficient for the *caspase-2* gene (*Casp2*^*−/−*^) do not develop spontaneous age-related tumors^19^, they show enhanced genomic instability and increased tumorigenesis in different mouse models, including *EμMyc* lymphomas^7,20^, *Atm*^*−/−*^thymomas^8^; *MMTV/c-neu* mammary tumors^11^, *K-Ras*-induced lung tumors^10^, and DEN-induced hepatocellular carcinoma (HCC)^21^. Recent studies suggest that caspase-2 mediates its tumor suppressor function by inducing senescence and/or apoptosis of aneuploid cells^22,23^. As a consequence, loss of *caspase-2* leads to the survival of abnormal, multinucleated, and aneuploid cells, which are features of mitotic catastrophe and susceptibility to tumorigenesis^15,21,22^.

Correlative evidence supports a tumor suppressor function for caspase-2. The human *CASP2* gene on Ch7q34, is part of a region frequently deleted in hematological malignancies^24^. In addition, reduced *CASP2* expression has been reported in lymphoma and leukemia and correlates with poor prognosis in AML and ALL^25,26^. Somatic mutations in *CASP2*, although rare, are found in cases of high-grade colon and gastric cancer, lung, skin, and breast cancer^19,27,28^. Furthermore, in colorectal cancer reduced *CASP2* expression is caused by loss of its transcriptional regulator *BCL9L*, and is a key cause of aneuploidy tolerance, tumor progression, and resistance^29^. In contrast, *Casp2* deficiency in mice does not affect tumor onset or progression following 3-methylcholanthrene (3-MC)-induced fibrosarcoma or irradiation-driven lymphoma^20^. Our previous studies have also demonstrated that *Casp2* loss delays neuroblastoma development in the *Th-MYCN* mouse model, and that low *CASP2* expression correlates with increased survival in human neuroblastoma^30^. These findings suggest that caspase-2 has a context/tissue-specific function in tumor suppression and that both tissue and genomic variability can cooperate with caspase-2 to determine tumor outcome. To understand how caspase-2 mediates its differential functions in tumor suppression, it is important to determine if disruption of additional genes co-operate with caspase-2 in tissue-specific contexts.

In this study, we comparatively analyzed the transcriptomes of *Casp2*^*−/−*^ tumors from *EμMyc* and *Th-MYCN* mice to identify changes in transcriptional tumor networks that are influenced by *caspase-2* deficiency. While we identified several unique genes that were aberrantly expressed in the *Casp2*^*−/−*^ tumors, we found that *Megf6/EGFL3* was the only gene that was differentially expressed in the two tumor types. Our study also identified several enriched pathways and gene signatures specific to *EμMyc/Casp2*^*−/−*^ or *Th-MYCN/Casp2*^*−/−*^ tumors that may be associated with enhanced *EμMyc-*induced lymphomas and/or delayed *Th-MYCN*-mediated neuroblastoma. The cross-tumor-specific transcriptional aberrations are highly indicative of distinct roles for caspase-2 during neuronal and B-cell development that perhaps influence its tumor suppressor function.

## Results

### RNA-seq analysis of *Th-MYCN* and *EμMyc* tumors from *Casp2*^*−/−*^ mice

**Fig. 1:**
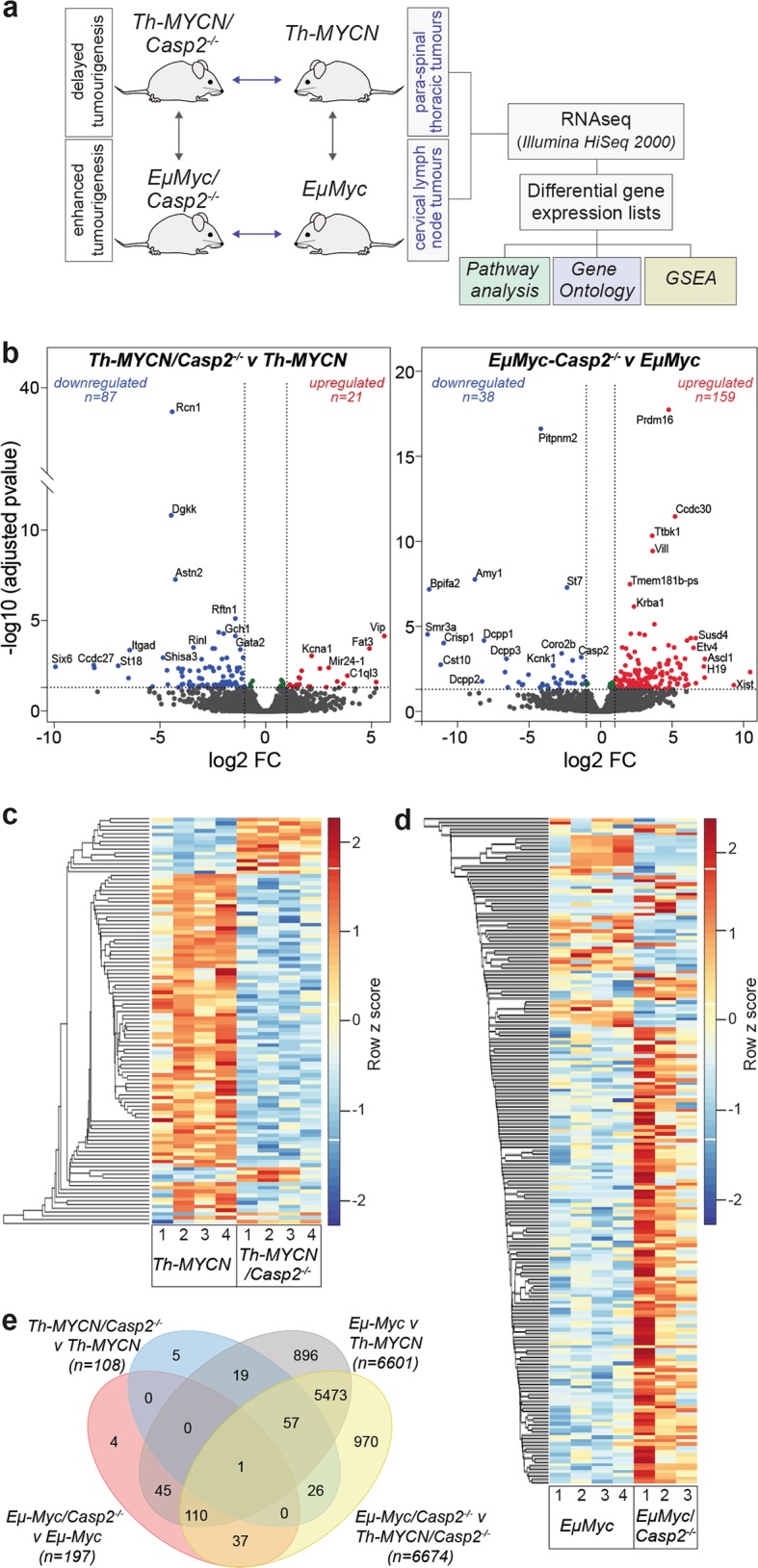
Experimental design and transcriptome profile of tumors from *Th-MYCN/Casp2*^−/−^ and *EµMyc/Casp2*^−/−^ mice. **a** Experimental design for RNA-seq and analysis of differentially expressed genes (DEGs) from tumor tissue. **b** Volcano plot illustrating DEGs in a comparison of *Th-MYCN*/*Casp2*^*−/−*^ and *Th-MYCN* tumor samples (top) or *EµMyc/Casp2*^*−/−*^ and *EµMyc* tumor samples. Colored points represent all DEGs (cut-off FDR < 0.05) with fold change > 2, that are either overexpressed (red) or underexpressed (blue) in *Casp2*^*−/−*^ compared with WT tumor counterparts. For a complete list of DEGs, see Supplementary Tables S2a and S2b. **c**, **d** Heat-maps of DEGs when comparing **c** *Th-MYCN*/*Casp2*^*−/−*^ and *Th-MYCN* and **d** *EµMyc/Casp2*^*−/−*^ and *EµMyc* tumor transcriptomes. Heat-maps display the number of increased (red) or decreased (blue) genes. For gene lists associated with heat-maps see Supplementary Tables S3a and S3b. **e** Four-way Venn diagram summary of unique and overlapping DEGs in the indicated comparison groups. The list of unique and overlapping genes in each group is provided in Supplementary Table S4a

To identify the transcriptomic differences in tumors from *Casp2*^*−/−*^ mice that are associated with delayed *Th-MYCN* induced neuroblastoma^30^ and/or enhanced *EμMyc* lymphoma development^7^, we carried out RNA sequencing on tumor tissue isolated from *Th-MYCN* and *Th-MYCN/Casp2*^*−/−*^ or from *EμMyc* and *EμMyc/Casp2*^*−/−*^ mice (Fig. 1a, Supplementary Tables S1a-b). Multidimensional scaling plots showed that the *EμMyc* and *Th-MYCN* tumor transcriptomes form distinct clusters (Supplementary Figure S1a), highlighting differences in gene expression changes between the two tumor types.

Comparisons of the differentially expressed genes (DEGs) were performed between (i) WT and *Casp2*^*−/−*^ tumors to identify genes associated with tumorigenesis following *Casp2* loss and (ii) between the *EμMyc/Casp2*^*−/−*^ and *Th-MYCN/Casp2*^*−/−*^ tumor samples, to narrow down candidate genes affected by *Casp2* deficiency and/or that enhance lymphomagenesis in *EμMyc/Casp2*^*−/−*^ mice. These DEGs are summarized as volcano plots (Fig. 1b, Supplementary Tables S2a-f). From the heat-maps, it is clear that *Casp2* deficiency changes the expression of many genes, with more downregulated genes in *Th-MYCN/Casp2*^*−/−*^ compared to *Th-MYCN* tumors (Fig. 1c) and more upregulated genes in *EμMyc/Casp2*^*−/−*^ versus *EμMyc* tumors (Fig. 1d, Supplementary Tables S3a-b).

In total, 13,714 genes were included for analysis in each comparison group (Supplementary Tables S2a-f). Of these, 108 DEGs were identified in the *Th-MYCN/Casp2*^*−/−*^ versus *Th-MYCN* tumor comparison and 197 DEGs in the *EμMyc/Casp2*^*−/−*^ versus *EμMyc* tumors (fold change [FC] > 2 and < −2; FDR < 0.05). As expected, there were extensive differences in gene expression when comparing the two different tumor types, including 6601 DEGs when comparing *EμMyc* to *Th-MYCN* tumors and 6674 DEGs in the *EμMyc/Casp2*^*−/−*^ versus *Th-MYCN/Casp2*^*−/−*^ tumor comparison. A 4-way Venn diagram identified several exclusive and common DEGs between each comparison group (Fig. 1e, Supplementary Table S4a). In particular, the *Th-MYCN/Casp2*^*−/−*^ comparison identified four unique downregulated genes (*Ncor2, Diras2, Ednrb, Shb*) and one upregulated *(Eya2*) gene, with roles in transcription (*Ncor2*), signal transduction (*Ednrb, Shb*), melanogenesis (*Ednrb*), and DNA damage repair (*Eya2*). Two of the unique *EμMyc/Casp2*^*−/−*^ genes have associated roles in transcription regulation (*Six5*) and calmodulin binding (*Nrgn*) and have been previously associated with lung squamous cell carcinoma, breast cancer (*Six5*^31,32^), and T-cell lymphoma (*Nrgn*^33^). *Tdrd5* was excluded as it was only increased in one out of three biological replicates. Significant downregulation of *Casp2* was also verified in the *EμMyc/Casp2*^*−/−*^ comparison and *Casp2* gene deletion was confirmed by genotyping and immunoblotting in all tumor samples.

**Fig. 2:**
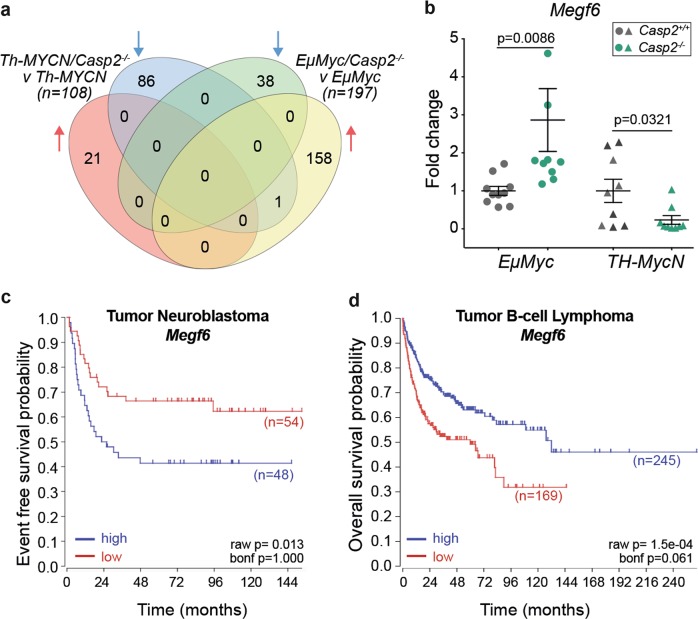
Comparison of upregulated and downregulated genes in *Th-MYCN/Casp2*^−/−^and *EµMyc/Casp2*^−/−^tumors. **a** Four-way Venn diagram summary illustrating common and exclusive upregulated and downregulated genes when comparing *Th-MYCN*/*Casp2*^*−/−*^ versus *Th-MYCN* and *EµMyc/Casp2*^*−/−*^ versus *EµMyc* tumor transcriptomes (FC > 2 and < −2; FDR < 0.05). Complete gene lists are provided in Supplementary Table S4b. **b** Quantitative PCR analysis of differentially expressed *Megf6* gene from the indicated tumor groups. Values represent means ± S.E.M, with *P*-values indicated (*n* = 9/group). **c**, **d** Kaplan–Meier plots showing the association of *Megf6* expression with patient survival in Neuroblastoma (**c**) and B-cell lymphoma (**d**)

To identify common genes that are significantly altered in the *Casp2*^*−/−*^ samples, the DEGs were subdivided into upregulated and downregulated lists, illustrated in a Venn diagram (Fig. 2a). This identified 86 uniquely downregulated and 21 upregulated genes in *Th-MYCN/Casp2*^*−/−*^ versus *Th-MYCN* tumors and 38 downregulated and 158 upregulated genes in *EμMyc*/*Casp2*^*−/−*^ versus *EμMyc* tumors (Fig. 2a, Supplementary Table S4b). There was also a single overlapping gene; *Megf6* (Multiple EGF-Like Domains 6) differentially altered in *EμMyc*/*Casp2*^*−/−*^ and *Th-MYCN/Casp2*^*−/−*^ tumors, and this was validated by quantitative PCR in additional tumor samples (Fig. 2b).

We used R2: Genomics Analysis and Visualization Platform, to examine the correlation between *Megf6* transcript expression and clinical outcome in various human neuroblastoma and B-cell lymphoma publicly available expression array data sets. Our previous data showed that *Casp2* levels correlated with clinical outcome in a subset of *MYCN* non-amplified human neuroblastomas^30^. Here, we also found that higher *Megf6* transcript levels are associated with poor outcome in this neuroblastoma subset (Fig. 2c), and this is consistent with our RNA-seq data and the delayed tumorigenesis observed in the *Th-MYCN/Casp2*^*−/−*^ mouse model^30^. We did not find any significant association in *MYCN*-amplified neuroblastoma. In contrast, we found there was a trend for lower *Megf6* expression being predictive of poorer B-cell lymphoma outcome (e.g., Xiao – 420, fRMA-u133p2 dataset; *P* = 0.061) (Fig. 2d). Notably, high *Megf6* expression in B-cell lymphomas also showed a somewhat poor survival outcome, indicating that *Megf6* levels are probably not predictive of lymphoma progression.

### Functional enrichment analysis of genes in *Th-MYCN/Casp2*^*−/−*^ tumors

**Fig. 3:**
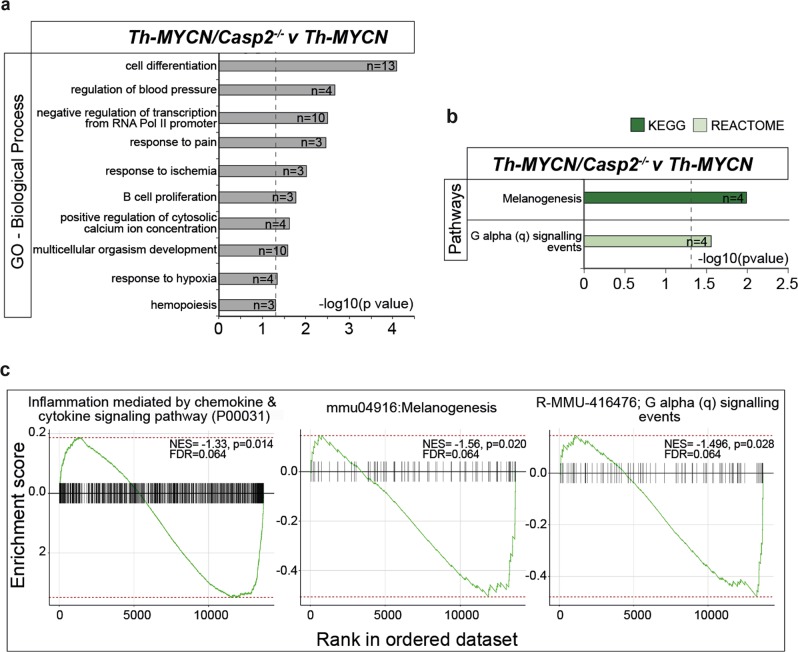
Functional category enrichment analysis of differentially expressed genes in *Th-MYCN/Casp2*^−/−^tumors. **a** Gene Ontology annotation analysis summary of enriched biological processes associated with unique downregulated genes in *Th-MYCN*/*Casp2*^*−/−*^ tumors (*n* = 87). Complete lists are provided in Supplementary Table S5a. **b** Pathway enrichment analysis (KEGG and REACTOME), of the differentially expressed downregulated genes in *Th-MYCN*/*Casp2*^*−/−*^ tumors (*n* = 87). Complete lists are provided in Supplementary Table S6a. The number of genes associated with each biological process and pathway is indicated. Dotted line indicates significance cut off (-log10 (*P*-value), Fisher's exact test). **c** Illustration of GSEA score curves. Enrichment plots are shown for the identified upregulated and downregulated pathways from (**b**) in the *Th-MYCN*/*Casp2*^*−/−*^ tumor comparison group. Black bars represent the position of members of the category in the ranked list together with the running enrichment score (plotted in green). NES = normalized enrichment score, FDR = false discovery rate

We next identified significantly altered gene ontology processes that were enriched in *Casp2*^*−/−*^ tumors, compared to their WT counterparts. We firstly analyzed the DEGs in the *Th-MYCN/Casp2*^*−/−*^ versus *Th-MYCN* tumor comparison. While there were no detected ontology terms associated with the small upregulated gene list (*n* = 21), the 87 downregulated genes were associated with 10 categories of ‘Biological Process’ (BP), 4 categories of ‘Molecular Function’ (MF), and 9 categories of ‘Cellular Component’ (CC) (*P* < 0.05, Fisher’s exact test) (Fig. 3a, Supplementary Table S5a). The top BP was cell differentiation (GO:0030154) (Fig. 3a, Supplementary Table S5a), with many of these genes also being associated with the BPs of transcriptional regulation from RNA polymerase II promoter (GO:0000122) and multicellular organismal development (GO:0007275). This indicates that *Casp2* loss can affect neuronal differentiation and gene transcription in neuroblastoma. Of note, response to hypoxia (GO:0001666) and ischemia (GO:0002931), processes previously associated with caspase-2 function^34–37^ were also enriched.

KEGG pathway analysis identified melanogenesis as the only enriched pathway, associated with four downregulated genes (*Ednrb, Mitf, Lef1, Camk2a*) (Fig. 3b, Supplementary Table S6a). Further analysis using REACTOME and PANTHER classification systems also identified G-alpha (q) signaling events (*P* = 0.03), inflammation mediated by chemokine and cytokine signaling pathway (*P* = 0.018) (Supplementary Table S6a). Gene set enrichment analysis (GSEA)^38^, using the gene list ranked by the FDR score, confirmed significant enrichment of each of these pathways (Fig. 3c).

### Functional enrichment analysis of genes in *EμMyc/Casp2*^*−/−*^ tumors

**Fig. 4:**
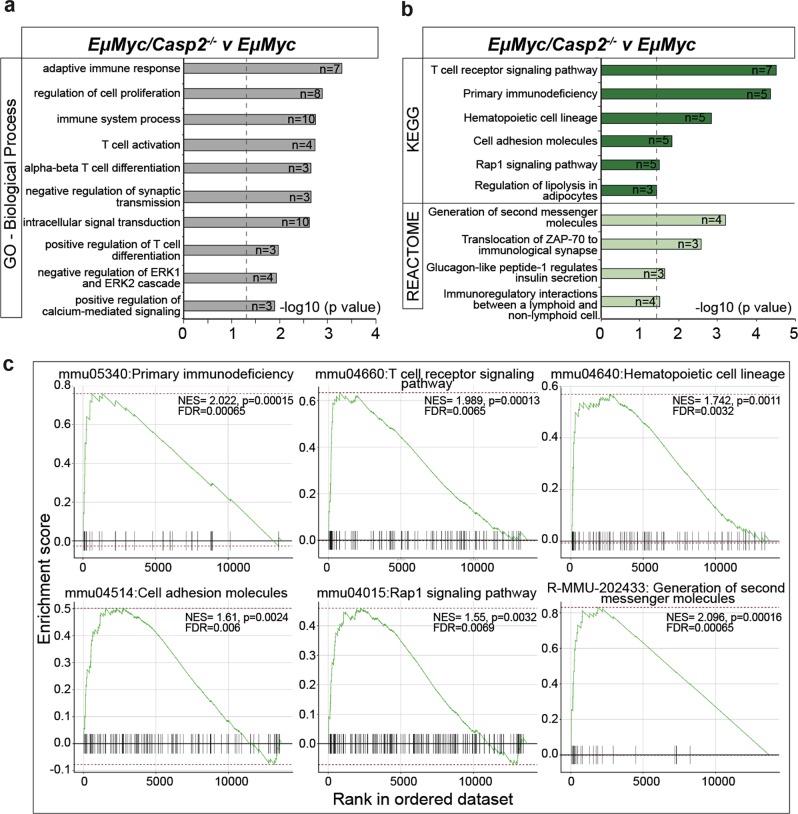
Functional category enrichment analysis of differentially expressed genes in *EµMyc/Casp2*^−/−^tumors. **a** Gene Ontology annotation analysis summary of the top 10 enriched biological processes associated with unique upregulated genes in *EµMyc/Casp2*^*−/−*^ tumors (*n* = 159). Complete lists are provided in Supplementary Table S5b. **b** Pathway enrichment analysis (KEGG and REACTOME), of the differentially expressed upregulated genes in *EµMyc/Casp2*^*−/−*^ tumors (*n* = 159). Complete lists are provided in Supplementary Table S6b. The number of genes associated with each biological process and pathway is indicated. Dotted line indicates significance cut off (-log10 (*P*-value), Fisher's exact test). **c** Illustration of gene set enrichment analysis (GSEA) score curves. Enrichment plots are shown for the identified upregulated and downregulated pathways from the *EµMyc/Casp2*^*−/−*^ tumor comparison group. Black bars represent the position of members of the category in the ranked list together with the running enrichment score (plotted in green). NES = normalized enrichment score, FDR = false discovery rate

Analysis of GO terms in the *EμMyc/Casp2*^*−/−*^ versus *EμMyc* tumor comparison identified several BPs associated the 159 upregulated genes, including immune response (GO:0002250, GO:0006955), immune system process (GO:0002376), T-cell activation (GO:0042110), T-cell differentiation (GO:0046632, GO:0045582, GO:0033077, GO:0030217), and inflammatory response (GO:0006954) (Fig. 4a, Supplementary Table S5b), indicative of a significant immune responses in *EμMyc/Casp2*^*−/−*^ tumors. Increased processes commonly associated with tumorigenesis including signal transduction (GO:0035556, GO:0007165, GO:0009966, GO:0007169, GO:0070373), cell proliferation (GO:0042127, GO:0008285), and negative regulation of cell adhesion (GO:0007162) were also enriched (Fig. 4a and Supplementary Table S5b).

Consistent with the enriched BPs, KEGG pathway analysis identified T-cell receptor signaling and primary immunodeficiency as the most significantly altered pathways followed by cell adhesion molecules and Rap1 signaling (Fig. 4b, Supplementary Table S6b). All pathways were significantly enriched when analyzed using GSEA (Fig. 4a, c), suggesting that caspase-2 may have a role in regulating T-cell signaling and/or Rap1 signaling to influence lymphoma development in *EμMyc* mice.

### Differential expression of genes in *caspase-2* deficient tumors associated with increased survival in human neuroblastoma

**Fig. 5:**
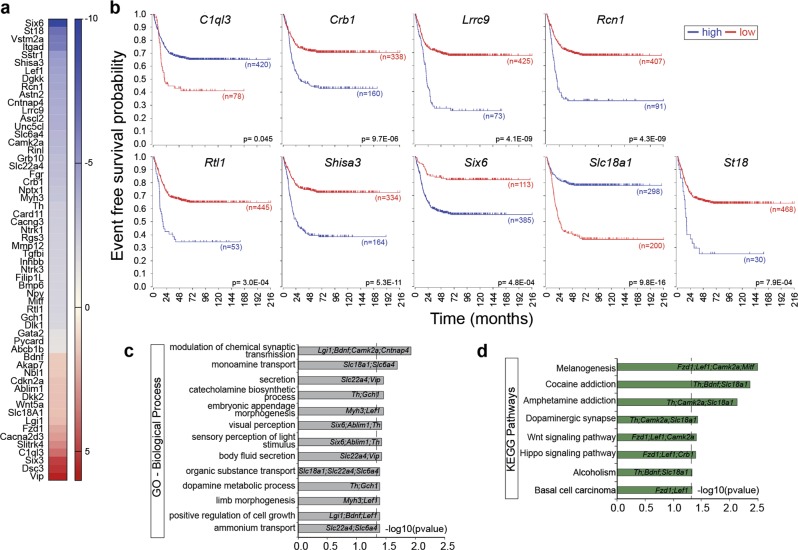
Differentially expressed genes in *Th-MYCN/Casp2*^−/−^tumors associated with survival in neuroblastoma patients. **a** Heat-map illustrating the increase (red) and decrease (blue) in expression (log2-fold change) of various neuroblastoma-associated genes in *Th-MYCN/Casp2*^*−/−*^ tumors compared to *Th-MYCN* tumors. Gene lists and pathway associations are available in Supplementary Table S7a. **b** Event-free survival curves for neuroblastoma patients from publicly available expression array data showing the expression correlation of the indicated genes with survival outcome in neuroblastoma. *P*-values using the method of Kaplan–Meier (Bonferroni correction) are shown. **c** Gene Ontology annotation analysis of the significantly enriched biological processes (Benjamini adjusted *P*-value < 0.05), associated with the genes listed in (**a**), that show altered gene expression (FDR < 0.1) and are strongly associated with neuroblastoma outcome in (**b**) (*n* = 41). Complete gene ontology lists are provided in Supplementary Table S7c. **d** Pathway enrichment analysis (KEGG) associated with neuroblastoma-associated genes above (*n* = 41). Complete pathway enrichment lists are provided in Supplementary Table S7d. The genes associated with each biological process and pathways are indicated. Dotted line indicates significance cut off [-log10(*P*-value), Benjamini corrected]

To identify genes associated with delayed neuroblastoma development in *Th-MYCN/Casp2*^*−/−*^ mice, we examined various molecular biomarkers of neuroblastoma development and prognosis^39,40^. The expression levels of several favorable neuroblastoma genes were found to be significantly altered in *Th-MYCN/Casp2*^*−/−*^ tumors, including increased expression of *Slc18A1*, *Vip*, and reduced expression of *Gata2*, *Gch1*, *Fgr*, and *Six6*. While the decrease in tyrosine hydroxylase (*Th*) expression was not significant in the biological replicates (FDR =0.584) (Fig. 5a, Supplementary Table S7a), we did note that several genes adjacent to *Th* on chromosome 7 (*Ascl2*, *Igf2*, and *H19*) were also somewhat decreased (Supplementary Table S7b). This suggests this gene region may be affected in some cells.

To determine the correlation between the expression of these genes and neuroblastoma survival, we analyzed data from published studies along with publicly available expression array data sets from primary human neuroblastoma tumor samples (R2 database). Overall, we found that downregulation of 7 genes (*Crb1, Lrrc9, Rcn1, Rtl1, Shisa3, Six6, St18*) and upregulation of 2 genes (*C1ql3, Slc18A1*), are strongly predictive of favorable neuroblastoma outcome (Fig. 5b, Supplementary Table S7a), consistent with delayed tumor development in *Th-MYCN/Casp2*^*−/−*^ mice^30^.

Our analysis also identified altered expression of several known neuroblastoma-associated genes that would be predictive of unfavorable neuroblastoma outcome (Supplementary Figure S3b, Supplementary Table S7a). These include decreased levels of *Ntrk1*, *Ntrk3* that are associated with poorly differentiated tumors and poor prognosis in neuroblastoma^40,41^ (Fig. 5a, Supplementary Table S7a). We also did not find significant changes in other neuroblastoma marker genes (e.g., *Bcl2, Cd44, Dcc, Ddx1, Lmo1, Max, Mdr1/Abcb1, Mrp/ Abcc1, Nf1, Ngf, Ntf3, Ntrk2, Nme1, Odc1*, *Rb1, Trp53*)^39^ (Supplementary Table S2a). These findings importantly show that these genes do not necessarily have a role in delayed tumor onset in *Th-MYCN/Casp2*^*−/−*^ and/or their role in neuroblastoma onset may be counteracted by loss of *Casp2*.

**Fig. 6:**
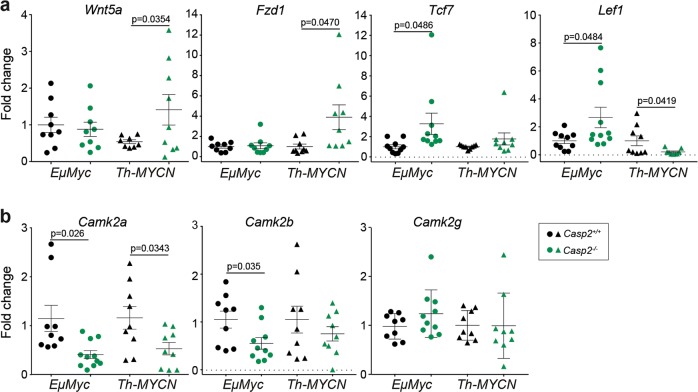
Validation of the differential expression of Wnt pathway associated genes in *Casp2*^−/−^ tumors. Quantitative PCR analysis of the Wnt pathway associated genes *Wnt5a, Fzd1, Tcf7,* and *Lef* (**a**) and CamkII family members *Camk2a, Camk2b, Camk2g* (**b**) in *EµMyc/Casp2*^*−/−*^ and *Th-MYCN*/*Casp2*^*−/−*^ tumors as indicated. Values represent mean ± S.E.M, with *P*-values indicated (*n* = 8–11/group)

Gene ontology analysis of the significantly altered neuroblastoma genes indicated their involvement in synaptic transmission, monoamine transport and cell growth regulation (Fig. 5c, Supplementary Table S7b). Interestingly, many of these genes are associated with melanogenesis, dopaminergic synapse, Wnt, and Hippo signaling (Fig. 5d, Supplementary Tables S7a and S7c). Downregulation of melanogenesis is associated with reduced expression of the transcription factor *Mitf*, along with Wnt signaling pathway components (*Fzd1*, *Lef1*, *Camk2a*). We noted a trend of altered expression of other Wnt signaling components (*Wnt5a*, *Dkk2*, *Bmp6*; FDR > 0.1) and their regulators, including *Gata2* and its target homeobox transcription factors *Six3* and *Hesx1* (Supplementary Tables S2a, S7a), which also have roles in neural determination^42,43^. Increased expression of *Wnt5a and Fzd1* and decreased expression of *Lef1* and *Camk2a* were validated in different tumor samples by quantitative PCR (Fig. 6a, b). Together, these data identify altered expression levels of several Wnt-associated genes and suggest a fine-tuning of Wnt signaling components in *Th-MYCN/Casp2*^*−/−*^ tumors that may determine neural crest cell (NCC) specification to influence neuroblastoma onset.

### Differential expression of genes in *caspase-2* deficient tumors associated with enhanced *EuMyc* lymphoma

To identify candidate oncogenes and tumor suppressor genes differentially expressed in the *EμMyc/Casp2*^*−/−*^ tumors that may contribute to enhanced lymphomagenesis, we interrogated several published cancer gene lists^44–46^ and databases (Sanger Cancer Gene Consensus; Bushman Lab ‘*allOnco*’ list; Oncogene Tumor Suppressor Gene Resource). This analysis identified a 147-gene signature in the *EμMyc/Casp2*^*−/−*^ tumors that included increased expression of a set of 72 oncogenes, decreased expression of nine genes with known or predicted tumor suppressor functions and 12 genes reported to have both oncogenic and tumor suppressor functions (Figure S7a, Supplementary Table S8a). Many oncogenes have previously been associated with *EμMyc*-mediated tumor progression and/or B-cell lymphoma^47–50^. Several of these genes are clustered on nearby chromosome regions, including Ch.4 (*Megf6-Prdm1, Arhgef16*), Ch.6 (*Cd4*-*Lag3, Cd8b*-*Cd8a*), Ch.9 (*Cd3d, Cd3g*, *Cd3e*), Ch.10 (*Dgka*-*Pme1*), and Ch.11 (*Sgsh, Slc26a11, Card14*) (Supplementary Table S8b) indicative of possible common regulatory elements or amplification of these regions in the *EμMyc/Casp2*^*−/−*^ tumors. These findings highlight changes in the expression of multiple cancer-associated genes and/or gene regions that likely contribute to enhanced *EμMyc* lymphomagenesis onset.

**Fig. 7:**
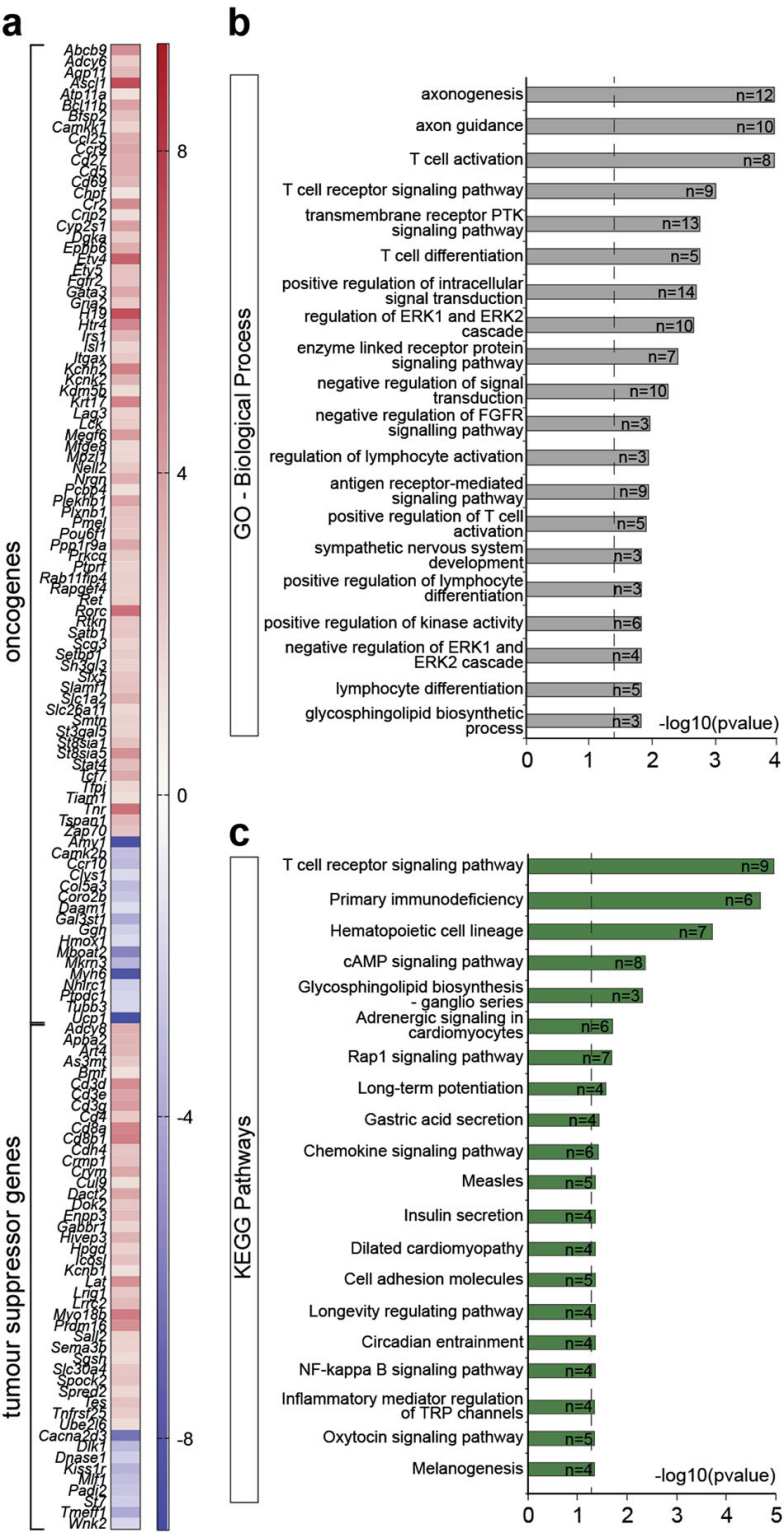
Differentially expressed genes in *EµMyc/Casp2*^−/−^ tumors associated with cancer progression. **a** Heat-map illustrating the increase (red) and decrease (blue) in expression (log2-fold change) of various cancer-associated genes in *EµMyc/Casp2*^*−/−*^ tumors. **b** Gene Ontology annotation analysis of the top 20 significantly enriched biological processes (Benjamini adjusted *P* < 0.05), associated with the genes listed in (**a**), that show changes in gene expression (FDR < 0.1) (*n* = 147). The number of genes associated with each biological process is indicated. Complete gene ontology lists are provided in Supplementary Table S8c. **c** Pathway enrichment analysis (KEGG and REACTOME), of the cancer-associated genes differentially expressed in *EµMyc/Casp2*^*−/−*^ tumors (*n* = 147). The top 20 enriched pathways are shown. Complete enriched pathway lists are provided in Supplementary Table S8d. The genes associated with each pathway are indicated. Dotted line indicates significance cut off (-log10(*P*-value), Benjamini corrected)

The majority of the genes that make up the Myc core signature (MCS), associated with lymphoma malignancy and progression^51^ were not further altered in *EμMyc/Casp2*^*−/−*^ compared to *EμMyc* tumors (Fig. 7, Supplementary Table S8a). Interestingly, we found nine common upregulated genes, which form part of the *Bcor* expression signature in *EμMyc* mice^52^, (*Bcl6, Camkk1, Cr2, Hivep3, Kcnb1, Lef1, Slamf1, St3gal5, Ube2l6*) (Supplementary Table S8a). However, there was no evidence of an associated increase in TGFβ signaling components in *EμMyc/Casp2*^*−/−*^ tumors, suggesting that *Bcor*-associated TGFβ signaling may not contribute to enhanced lymphomagenesis in *EμMyc/Casp2*^*−/−*^ mice.

Analysis of the 147-gene signature in *EμMyc/Casp2*^*−/−*^ tumors, identified axonogenesis, T-cell signaling, protein kinase B signaling, and signal transduction as the top enriched BPs and MFs (Fig. 7b, Supplementary Table S8c). Consistent with this, T-cell receptor signaling, primary immunodeficiency and hematopoietic cell lineage were the top enriched KEGG pathways (Fig. 7c, Supplementary Table S8d), with Rap1 signaling and cell adhesion molecules also identified from our total DEG list (Fig. 4e). This indicates the DEGs in *EμMyc/Casp2*^*−/−*^ tumors are largely established cancer-associated genes.

Interestingly, the pathways melanogenesis and Wnt signaling were identified in the DEGs from both [*EμMyc/Casp2*^*−/−*^ v *EμMyc*] (Fig. 7c, Supplementary Table S8d) and [*Th-MYCN/Casp2*^*−/−*^ v *Th-MYCN*] (Fig. 5d) tumor comparisons. Increased expression of *Tcf7*, and its co-operating transcription factor *Lef1*, were further confirmed by quantitative PCR analysis in different *EμMyc/Casp2*^*−/−*^ tumor samples (Fig. 6a).

### Comparison of DEGs in *EμMyc/Casp2*^*−/−*^ compared to *Th-MYCN/Casp2*^*−/−*^ tumors

**Fig. 8:**
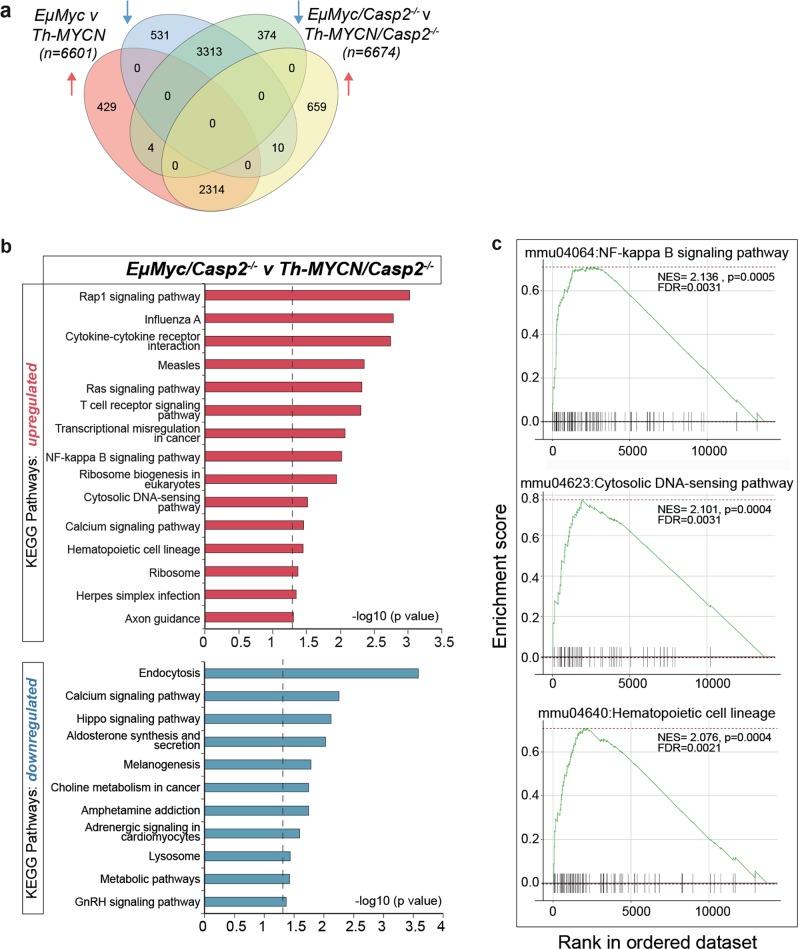
Comparison of differentially expressed genes and enriched pathways in *EµMyc/Casp2*^−/−^ and *Th-MYCN/Casp2*^−/−^ tumors. **a** Four-way Venn diagram summary of unique and overlapping upregulated and downregulated genes in the indicated comparison groups. The list of unique and overlapping genes in each group is provided in Supplementary Tables S4c and S4d. **b** KEGG Pathway enrichment analysis of upregulated (red) and downregulated (blue) pathways in *EµMyc/Casp2*^*−/−*^ tumors compared to *Th-MYCN*/*Casp2*^*−/−*^ tumors. Number of genes associated with each pathway is indicated. Complete enriched pathway lists are provided in Supplementary Table S6d. Dotted line indicates significance cut off (-log10(*P*-value) Fisher's exact test). **c** GSEA of the upregulated and downregulated pathways from (**b**). Black bars represent the position of members of the category in the ranked list together with the running enrichment score (plotted in green). NES = normalized enrichment score, FDR = false discovery rate

We compared the DEGs from *EμMyc/Casp2*^*−/−*^ to *Th-MYCN/Casp2*^*−/−*^ tumors in aim to define unique components and pathways associated with enhanced *EμMyc*-mediated lymphomagenesis. A 4-way Venn diagram identified 659 uniquely upregulated and 374 downregulated genes in the *EμMyc/Casp2*^*−/−*^ compared to *Th-MYCN/Casp2*^*−/−*^ tumors (Fig. 8a). An additional 14 genes (*Abcc2, Ccdc27*, *H19, Itgax, Krt17, Megf6, Mitf, Mmp12, Rcn1, Rorc and Amy1, Col5a3, Kcnk1, Pitpnm2*) were differentially regulated compared to the *EμMyc* v *Th-MYCN* DEGs (Supplementary Table S4c), with some genes significantly associated with extracellular matrix organization (*Mmp12, Col5a3, Itgax*) (GO:0030198, Benjamini corrected *P* = 0.047), highlighting an important difference in the *EμMyc/Casp2*^*−/−*^ tumors.

Gene ontology analysis of the exclusively upregulated genes (*n* = 659) identified the BPs immune response, inflammatory response, angiogenesis, and cell migration (Benjamini corrected *P* < 0.05) (Supplementary Figures S4a-b, Supplementary Tables S5c-d). Interestingly, regulation of apoptosis, response to hypoxia, reactive oxygen species, metabolic process and hematopoietic progenitor cell differentiation are processes consistent with loss of caspase-2 function^6^ (Supplementary Figure S4b, Supplementary Table S5d). Analysis of the downregulated genes (*n* = 374) identified 13 unique BPs, with neuron maturation being the most significantly decreased, perhaps consistent with its primary role in *Th-MYCN/Casp2*^*−/−*^ tumors (Benjamini corrected *P* < 0.05). Other notable downregulated pathways (e.g., response to fatty acid, ubiquitination, negative regulation of epithelial cell proliferation, and cell cycle arrest) highlight multiple uniquely deregulated processes, previously been linked to *Casp2* loss, and suggest their role in contributing to enhanced lymphomagenesis in *EμMyc/Casp2*^*−/−*^mice.

KEGG pathway analysis identified increased Rap1 signaling and cytokine-cytokine receptor interaction as the most significantly enriched and unique pathways (*P*<0.001, Fisher's exact test) (Fig. 8b, Supplementary Tables S6c-d). In addition to T-cell receptor signaling identified from the above analysis (Fig. 4b), enrichment of NFκB signaling and cytosolic-DNA sensing were also confirmed by GSEA (Fig. 8c) (Supplementary Table S6d). Interestingly, endocytosis was the top pathway associated with downregulated genes (Benjamini corrected *P* = 0.049), a process that has emerged as a key hallmark of cancer, by affecting cell surface expression of critical molecules and signaling processes^53^. Calcium signaling, and Hippo signaling pathways were also uniquely enriched (Fig. 8b, Supplementary Table S6d). Consistent with this, reduced expression of *Camk2a* and *Camk2b* was validated in different *EμMyc/Casp2*^*−/−*^ tumor samples (Fig. 6b). These findings highlight several new pathways that are deregulated in *EμMyc/Casp2*^*−/−*^ tumors that can contribute to tumorigenesis.

## Discussion

We used transcriptome profiling to characterize gene expression changes and pathways affected by *Casp2* deficiency, in tumors from *Th-MYCN* neuroblastoma^30^ and *EμMyc* lymphoma transgenic mouse models^7^. Our study has identified specific caspase-2 expression signatures in the different tumor types that likely contribute to the distinct tumor outcomes^7,30^. The diversity in gene expression highlights the (1) heterogeneity in tumor cell populations and/or clonal co-operation between sub-clones within the same tumor (2) tumor type-specificity (3) aberrations in components of several different signaling pathways, and/or (4) subtle differences that make it difficult to detect single causative pathways.

While the changes in gene expression between the *Th-MYCN/Casp2*^*−/−*^ and *EμMyc/Casp2*^*−/−*^ comparison groups were distinct, it was interesting that *Megf6* was the only gene differentially expressed in the *Casp2*^*−/−*^ tumors. Megf6 is implicated in neural system disorders, such as ataxia^54^ and recently, the epithelial-to-mesenchyme transition (EMT) to promote metastasis in colorectal cancer via TGFβ signaling^55^. Consistent with a tumor progression function, we found higher expression of *Megf6* in *EμMyc/Casp2*^*−/−*^ and lower expression in *Th-MYCN/Casp2*^*−/−*^ tumors. Lower levels of *Megf6* expression also correlated with better survival outcome in human neuroblastoma, suggesting it may contribute to delayed neuroblastoma in the *Th-MYCN/Casp2*^*−/−*^ mouse^30^. The lack of clinical correlation with *Megf6* expression in B-cell lymphoma may be indicative of a tissue-specific function for Megf6 and/or that *Casp2* loss can cooperate with high *Megf6* expression to augment lymphomagenesis in the *EμMyc/Casp2*^*−/−*^ model. Nevertheless, these findings provide premise to further investigate Megf6 role and potential co-operation with caspase-2 function in tumorigenesis.

The delayed neuroblastoma onset in *Th-MYCN/Casp2*^*−/−*^ mice, previously highlighted the tissue/context specific role for caspase-2 in tumor suppression^30^. Our study has now identified cell differentiation as the most significantly altered biological process in *Th-MYCN/Casp2*^*−/−*^ tumors, associated with downregulation of genes that regulate neuronal differentiation and cell fate determination (*Ascl2, Gldn, Mitf, Notch3, Shb, Syt17*) and many being transcription factors (*Ascl2, Batf3, Cbfa2t3, Elf4, Gata2, Mitf*). A role for caspase-2 in neuronal differentiation, remodeling and protection against ischemic injury, has been previously implicated^34,56,57^. Silencing *Casp2* can increase expression of neuronal differentiation markers^57^, and increased differentiation in *Casp2*^*−/−*^ neurons, would be consistent with favorable neuroblastoma outcome^40^ and delayed tumor onset in *Th-MYCN/Casp2*^*−/−*^ mice^30^. Increased neuronal differentiation is also associated with high ROS levels^58^, a feature of *Casp2*^−/−^ cells^14,15^, that can further contribute to neuronal differentiation^58^. Identification of caspase-2 substrate(s) and/or interacting partners in differentiating NCCs will be key to defining its role in neurons and elucidate its function in neuroblastoma.

Our analysis identified a 41-gene signature in *Th-MYCN/Casp2*^*−/−*^ tumors, associated with neuroblastoma outcome, and involved in altered melanogenesis, Wnt and Hippo pathway signaling. Melanogenesis is known to be inhibited by excessive ROS^59^ and may therefore be affected by the increased oxidative stress and ROS in *Casp2*^*−/−*^ cells^14,15^. Melanogenesis is also associated with aggressive neuroblastoma, characterized by higher tyrosinase activity and associated increase in DOPA synthesis^60–62^. The key regulator of tyrosinase is *Mitf*, was significantly reduced in *Th-MYCN/Casp2*^*−/−*^ compared to *Th-MYCN* tumors, and may suggest a defect in DOPA synthesis in *Th-MYCN/Casp2*^−/−^ tumors, consistent with the reduction in Dopaminergic synapse signaling. In addition, Wnt signaling regulates melanogenesis by stabilization and signaling through Mitf^59^. While a role for Wnt signaling in neuroblast differentiation has been described^43,63^, it has diverse functions in cell proliferation, polarity and migration, so its role in neuroblastoma is likely complex^63^. Nevertheless, the finding here, show that loss of *Casp2* affects several components regulating the Wnt signaling pathway in neuroblastoma, that could impact neuronal differentiation and possibly neuroblastoma onset in *Th-MYCN/Casp2*^*−/−*^ mice.

Our previous studies demonstrated that loss of *Casp2* accelerates *EμMyc*-driven lymphomagenesis^7^. This study has now identified aberrant expression of 147 cancer-associated genes including increased expression of 72 oncogenes and reduced expression of 9 TSG in the *EμMyc/Casp2*^*−/−*^ tumor samples. While many of these genes may play co-operating roles with *Casp2* loss to enhance *EμMyc*-mediated lymphomagenesis, aberrant expression of some genes are likely a consequence of enhanced tumor growth caused by *Casp2* loss. Interestingly, the key pathways associated with *EμMyc/Casp2*^*−/−*^ tumors, was increased immune response, in particular T-cell activation and signaling pathways. Higher expression of *CD4*, *CD25/IL2ra* and *Lag3* in *EμMyc/Casp2*^*−/−*^ tumors, are indicative of activated regulatory T-cells (Tregs) at the tumor site, which are associated with both favorable and unfavorable prognosis in B-cell lymphomas^64^. They can facilitate tumor immune evasion by directly suppressing B-lymphoma cells, and also function to inactivate tumor specific CD4^+^/CD8^+^ T-cells, leading to an immunosuppressive network associated with increased T-cell tolerance and reduced T-cell mediated killing^64^. This is a key factor in uncontrolled tumor growth and poor clinical outcome in many solid tumors^64^. Several clinical studies have also described the presence of T-cell markers (including CD3, CD4, CD8) in diffuse large B-cell lymphoma (DLBCL), but the prognostic significance of this is not clear^65^. Our data are highly suggestive that the active immune response plays a key role in augmenting tumorigenesis in *EμMyc/Casp2*^*−/−*^ mice. A role for caspase-2 in regulating inflammasome signaling and the innate immune response has been previously reported^6^. However, it is also likely that the potential role of caspase-2 in hematopoietic cell differentiation, (e.g., myeloid cell differentiation)^66^ may contribute to immunosurveillance mechanisms that determine tumor outcomes.

The comparison of gene expression profiles in *EμMyc/Casp2*^*−/−*^ tumors to *Th-MYCN/Casp2*^*−/−*^ tumors identified a significant enrichment of genes associated with Rap1 signaling. Rap1 regulates cell adhesion; a process also enriched in the *EμMyc/Casp2*^*−/−*^ tumors, and perhaps indicates a link between these two processes. While Rap1 can promote tumor cell migration, invasion, and metastasis^67^, these features have not been observed in *EμMyc/Casp2*^*−/−*^ mice, indicating possible alternative roles for Rap1 signaling in *EμMyc/Casp2*^*−/−*^ tumors. This may include Rap1 roles in regulating cytoskeletal dynamics and/or cell proliferation, previously associated with loss of caspase-2 function^6,12^. Alternatively, Rap1 signaling plays diverse roles in hematopoietic cells, including lymphocyte activation, migration/trafficking, immunological synapse^67^, B-cell development, and self-tolerance^68^. While the specific and diverse functions of Rap1 are context dependent, a role for caspase-2 in regulating Rap1 signaling is highly relevant to lymphoma onset and progression and will be important to further define the contributing roles of Rap1 and caspase-2 in B-cell lymphomagenesis.

In summary, this is the first description of tissue specific and differential gene expression signatures associated with caspase-2 deficiency that can influence tumorigenesis. In particular, our findings indicating that loss of *Casp2* impacts neuronal differentiation, is likely a key contributor to delaying neuroblastoma onset and reduced adrenal tumor development in *Th-MYCN/Casp2*^*−/−*^ mice. In contrast, increased immune processes, including hematopoietic cell lineage determination, are potential effectors in *EμMyc/Casp2*^*−/−*^ lymphomagenesis. In addition, we have identified aberrant expression of several oncogenes and tumor suppressor genes that potentially co-operate with *Casp2* loss, to determine tumor outcome. These findings reinforce the distinct contribution of signaling pathways associated with tumor onset and progression in both *Th-MYCN/Casp2*^*−/−*^ and in *EμMyc/Casp2*^*−/−*^ mice.

## Methods

### Tumor samples

Lymphomas from *EuMyc* and *EuMyc/Casp2*^*−/−*^ mice were taken from cervical lymph nodes^7^. Tumor samples from *Th-MYCN* and *Th-MYCN/Casp2*^*−/−*^ mice were selected from paraspinal thoracic tumors based on absence or reduced blood vascularization^30^. Four individual tumors per genotype were used for mRNA-seq analysis.

### RNA extraction

Total RNA was extracted from frozen tumor tissue using Trizol® reagent (Life Technologies) according to the manufacturer’s protocol. RNA was resuspended in diethylpyrocarbonate-(DEPC) treated water and quantified using a NanoDrop1000™ spectrophotometer (Thermo Scientific). RNA samples were quality tested using a 2100 Bioanalyser (Agilent Technologies) to confirm RNA integrity number (RIN) > 7.

### mRNA sequencing and bioinformatics analysis

RNA-seq and bioinformatics analysis was carried out at the ACRF Cancer Genomics Facility (Centre for Cancer Biology, UniSA). Briefly, polyA^+^ mRNA was enriched using oligo-dT beads and samples were barcoded for pooled sequencing on an Illumina HiSeq 2000 (Agilent Technologies). Short, single-end reads were carried out (1 × 50 bp flow cells) with four samples per lane. This yielded ~20–30 million raw reads per sample.

Sequence quality was analyzed using FastQC and the resulting data sets were aligned using the STAR alignment algorithm (https://www.ncbi.nlm.nih.gov/pubmed/23104886) to the UCSC mm10/GRCm38 version of the *Mus musculus* reference genome. Gene counts were obtained from the second strand of the STAR output and the resulting files were transferred to the R statistical environment. The edgeR Bioconductor package^69^ was used to fit a linear model and samples were log transformed for normalization and ranking and then expressed as log2-fold change of expression (Log2FC). Data are available in Gene Expression Omnibus (GEO) under accession GSE124051.

Multidimensional scaling plots were generated to explore the relationships in the data before and after processing. (Supplementary Figure S1a and Supplementary Table S1c). All biological replicates were similar to each other except for *EμMyc/Casp2*^*−/−*^ sample #212, which fell outside the *EμMyc* cluster. Normalization of data was able to correct for this, however a scatterplot matrix analysis of the *EμMyc* samples indicated that sample #212 was a strong outlier (Supplementary Figure S1a) and did not correlate well with other biological replicates (Supplementary Figure S1b). For this reason, *EμMyc/Casp2*^*−/−*^ sample #212 was removed from further differential expression analysis.

### Differential expression analysis of individual genes

Differential expression analysis of individual genes was carried out using edgeR. We divided samples into the two tumor types *EµMyc* and *MYCN* groups, and also into two genotype groups *Casp2*^*+/+*^ and *Casp2*^*−/−*^. Four-way comparisons in gene expression levels were made, including: (1) *Th-MYCN/Casp2*^*−/−*^ versus *Th-MYCN;* (2) *EuMyc/Casp2*^*−/−*^ versus *EuMyc;* (3) *EuMyc* versus *Th-MYCN*, and (4) *EuMyc/Casp2*^*−/−*^ versus *Th-MYCN/Casp2*^*−/−*^. Lastly we carried out an additional analysis of the differentially expressed factors caused by *Casp2* loss in *EµMyc* compared to *Th-MYCN* tumors; (*EµMyc* v *EµMyc/Casp2*^*−/−*^) versus (*Th-MYCN* v *Th-MYCN/Casp2*^*−/−*^). Raw counts were extracted for each of these samples and edgeR was used to calculate the DEGs between the two phenotypic groups and expressed as log2-fold change (Log2FC) of expression between conditions being tested.  Significant changes in gene expression levels were determined using adjusted *P*-values according to the Benjamini–Hochberg method to control for the false discovery rate (FDR) (Supplementary Tables S1c and S2a-f). For heat-maps, samples were clustered based on gene expression levels and false discovery rate (FDR < 0.05), and the most variable genes plotted (Fig. 1c, d, Supplementary Figures S2d-f, Supplementary Tables S3a-e).

### Enrichment analysis of biological processes and pathways

Analysis of Gene Ontology and enriched biological pathways was carried out on DEGs with log2FC > 1 and ≤ −1 (i.e., 2^(1); equivalent to an actual fold change of 2)^.^(FDR < 0.05), using the Functional Annotation Tool in the Database for Annotation, Visualization and Integrated Discovery (DAVID) Bioinformatics Resources 6.8 (https://david.ncifcrf.gov/home.jsp)^70,71^. Enriched pathways were analyzed from the KEGG (Kyoto Encyclopedia of Genes and Genomes) and REACTOME database in DAVID. The threshold value of gene counts was set at 3, and the EASE score was set at 0.1 and pathways with Fisher's exact *P* < 0.05, were considered to be enriched. Where indicated, pathways were also determined using PANTHER^72^ (http://www.pantherdb.org/about.jsp) and EnRichR (http://amp.pharm.mssm.edu/Enrichr/)^73,74^ databases. Gene Set Enrichment Analysis (GSEA) of pathways and genes was performed using Bioconductor fast pre-ranked GSEA (*fgsea*) package in R.

### Real-time quantitative PCR (qPCR)

Total RNA extracted from frozen tumor tissue, was reverse transcribed using the High Capacity cDNA Reverse Transcription Kit (Applied Biosystems) with MultiScribe™ Reverse Transcriptase and oligo-dT primers. Gene expression was normalized to the housekeeping gene β-actin and then expressed as fold change compared to *EµMyc* or *Th-MYCN*/*Casp2*^*+/+*^ tumor samples, using the 2^−∆∆Ct^ method. See Supplementary Table S9 for primer sequences.

### Statistical analyses

Statistical analysis was carried out using GraphPad Prism software (v 6.0, San Diego, CA, USA). Data are expressed as mean ± SEM and two-tailed unpaired *t*-test with Welch’s correction was used for pair-wise comparisons, unless otherwise indicated. Heat-maps were generated using R software, from natural log2 transformed data. Venn diagrams were generated using online software jvenn (http://jvenn.toulouse.inra.fr/app/example.html)^75^.

## Supplementary information

Supplementary Information

Supplementary figures

Supplementary Table S1

Supplementary Table S2

Supplementary Table S3

Supplementary Table S4

Supplementary Table S5

Supplementary Table S6

Supplementary Table S7

Supplementary Table S8

Supplementary Table S9
