# Supplementary material for: Transcriptome profiling of caspase-2 deficient EμMyc and Th-MYCN mouse tumors identifies distinct putative roles for caspase-2 in neuronal differentiation and immune signaling
Source: Cell Death Dis. 2019 Jan 22;10(2):56. doi: 10.1038/s41419-018-1296-0 (PMC6343006; doi:10.1038/s41419-018-1296-0)
Supplement: Supplementary file 1 — Supplementary Information [file 41419_2018_1296_MOESM1_ESM.docx]

**Supplementary Information**

**Supplementary Figure Legends**

**Supplementary Figure S1. Mulitdimensional Scaling (MDS) analysis of RNA-seq data.** (a) MDS plot from RNA-seq data shows distinct clustering of *EµMyc* (n=4) and *EµMyc/Casp2^-/-^*  (n=4) compared to *Th-MYCN* (n=4) and *Th-MYCN*/*Casp2^-/-^*  (n=4) tumor samples. (b) 2-dimensional scatterplot matrix of log2 normalized expression, illustrating the correlation between *EµMyc* (n=4) and *EµMyc/Casp2^-/-^*  (n=4) samples. Biological replicates within groups show little variation, except for *EµMyc/Casp2^-/-^* sample #212, which was omitted from further analysis due to poor correlation with other samples.

**Supplementary Figure S2. RNA-seq comparison of *EµMyc* and *Th-MYCN* tumor samples*.*** (a-c) Volcano plots illustrating differentially expressed genes when comparing (a) *EµMyc* and *Th-MYCN,* (b) *EµMyc/Casp2^-/-^* and *Th-MYCN*/*Casp2^-/-^* and (c) genes from [*EµMyc/Casp2^-/-^* v *EµMyc*] compared to [*Th-MYCN*/*Casp2^-/-^* v *Th-MYCN*]. Colored points represent differentially expressed genes (cut-off FDR<0.05) with log2FC >1 and <-1 that are either over-expressed (red) or under-expressed (blue) in each comparison group. For a complete list of differentially expressed genes, see Supplementary Table S2c and S2d. (d-f) Heat maps of differentially expressed genes associated with the comparison groups in a-c. Heat maps display the number of increased (red) or decreased (blue) genes. For gene lists associated with heat-maps see Supplementary Tables S3d - S3e.

**Supplementary Figure S3. Differential expression of genes in *Th-MYCN*/*Casp2^-/-^* associated with neuroblastoma prognosis.** Event-free survival curves for neuroblastoma patients from publicly available expression array data showing the expression correlation of the indicated genes with survival outcome in neuroblastoma. (a) Survival curves for genes where expression in *Th-MYCN*/*Casp2^-/-^* tumors correlates with favorable outcome and (b) Survival curves for genes where expression in *Th-MYCN*/*Casp2^-/-^* tumors correlates with unfavorable outcome. *P*values, using the method of Kaplan–Meier (Benjamini correction), are shown.

**Supplementary Figure S4. Gene ontology and pathway enrichment in *EµMyc* compared to *Th-MYCN* tumor samples*.*** (a, b) Gene Ontology annotation analysis of the significantly enriched biological processes (Benjamini adjusted *P*<0.05), associated with up-regulated (red) and down-regulated (blue) genes significantly altered in (a) *EµMyc* compared to *Th-MYCN* tumors and in (b) *EµMyc/Casp2^-/-^* compared to *Th-MYCN/Casp2^-/-^* tumors. Complete gene ontology lists are provided in Supplementary Table S5c. (c) Pathway enrichment analysis (KEGG and REACTOME) of up-regulated (red) and down-regulated (blue) genes in *EµMyc/Casp2^-/-^* compared to *Th-MYCN/Casp2^-/-^* tumors. The number of genes associated with each pathway is indicated. Complete enriched pathway lists are provided in Supplementary Table S6c.

**Supplementary Tables**

**Supplementary Table S1 (a-c):** Sequencing information including (a) sample summary and basic statistics, (b) raw counts and (c) normalized counts for each biological replicate (n=4 per genotype).

**Supplementary Table S2 (a-f):** Differentially expressed gene lists comparing tumor samples from the following genotypes: (a) *Th-MYCN/Casp2^-/-^* vs *Th-MYCN*, (b) *EμMyc/Casp2^-/-^* vs *EμMyc*, (c) *EμMyc* vs *Th-MYCN* and (d) *EμMyc/Casp2^-/-^* vs *Th-MYCN/Casp2^-/-^* comparisons and (e) an interaction comparing genes from (*EμMyc/Casp2^-/-^* vs *EμMyc*) to (*Th-MYCN/Casp2^-/-^* vs *Th-MYCN*). Table S2f provides a summary of genes that are significantly differentially expressed in at least one these comparisons. Log2FC= log2-fold change of expression between conditions being tested, LogCPM= average log2-counts per million; LR= likelihood ratio statistics; *P*value: exact p-value for differential expression; FDR= Benjamini adjusted *P*value.

**Supplementary Table S3 (a-e):** Differentially expressed gene lists corresponding to the order of the associated heat-maps displayed in Figure 1d and Supplementary Figure S2d-f, for the following comparisons: (a) *Th-MYCN/Casp2^-/-^* vs *Th-MYCN*, (b) *EμMyc/Casp2^-/-^* vs *EμMyc*, (c) *EμMyc* vs *Th-MYCN* and (d) *EμMyc/Casp2^-/-^* vs *Th-MYCN/Casp2^-/-^* and (e) an interaction comparing genes from (*EμMyc/Casp2^-/-^* vs *EμMyc*) to (*Th-MYCN/Casp2^-/-^* vs *Th-MYCN*).

**Supplementary Table S4 (a-c):** Common and exclusive gene lists identified by a four-way Venn diagram analysis of differentially expressed genes comparing (a) all DEGs from (*Th-MYCN/Casp2^-/-^* vs *Th-MYCN*); (*EμMyc/Casp2^-/-^* vs *EμMyc*,); (*EμMyc* vs *Th-MYCN*) and (*EμMyc/Casp2^-/-^* vs *Th-MYCN/Casp2^-/-^*), (b) up-regulated and down-regulated genes from (*Th-MYCN/Casp2^-/-^* vs *Th-MYCN*) and (*EμMyc/Casp2^-/-^* vs *EμMyc*,) and (c) up-regulated and down-regulated genes from (*EμMyc* vs *Th-MYCN*) and (*EμMyc/Casp2^-/-^* vs *Th-MYCN/Casp2^-/-^*) DEG lists. [FC>2 and <-2; FDR<0.05].

**Supplementary Table S5 (a-d):** Gene Ontology lists of unique up-regulated and down-regulated genes (FC>2 and <-2; FDR<0.05) for each comparison: (a) *Th-MYCN/Casp2^-/-^* vs *Th-MYCN*, (b) *EμMyc/Casp2^-/-^* vs *EμMyc*, (c) *EμMyc* vs *Th-MYCN* and (d) *EμMyc/Casp2^-/-^* vs *Th-MYCN/Casp2^-/-^*. Ontology terms include Biological Process (BP), Molecular Function (MF) and Cellular Component (CC) for each comparison, determined using DAVID Bioinformatics resources 6.8 ([http://david.abcc.ncifcrf.gov](http://david.abcc.ncifcrf.gov)).

**Supplementary Table S6 (a-d):** Lists of enriched biological pathways for differentially expressed and unique genes determined by Venn Diagram analysis (FC>2 and <-2; FDR<0.05) of up-regulated and down-regulated genes from: (a) *Th-MYCN/Casp2^-/-^* vs *Th-MYCN*, (b) *EμMyc/Casp2^-/-^* vs *EμMyc*, (c) *EμMyc* vs *Th-MYCN*, (d) *EμMyc/Casp2^-/-^* vs *Th-MYCN/Casp2^-/-^* comparisons. Pathways include Kyoto Encyclopedia of Genes and Genomes (KEGG) and REACTOME pathways identified using DAVID Bioinformatics resources 6.8 ([http://david.abcc.ncifcrf.gov](http://david.abcc.ncifcrf.gov)) and PANTHER (<http://www.pantherdb.org/pathway/>).

**Supplementary Table S7 (a-d):**

Differentially expressed genes in *Th-MYCN/Casp2^-/-^* compared to *Th-MYCN* tumors that are associated with known or predicted functions in Neuroblastoma. (a) Differentially expressed gene list with fold change (FC) and associated prognostic indicator, neuronal function and pathways. (b) Gene lists indicating chromosomal position on the mouse genome. (c) Gene ontology and (d) Pathway (KEGG, REACTOME and PANTHER) analysis of neuroblastoma associated genes (n=41) selected based on FC>2 and <-2 (*P*<0.05; FDR<0.1) and identified using EnrichR (<http://amp.pharm.mssm.edu/Enrichr/index.html>).

**Supplementary Table S8 (a-d):** Identification and analysis of cancer associated genes that are differentially expressed in *EμMyc/Casp2^-/-^* compared to *EμMyc* tumor samples. (a) Differentially expressed gene list with associated fold change (FC), oncogenic and/or tumor suppressor classification and pathways. (b) Gene list with associated chromosomal position on the mouse genome. (c) Gene ontology and (d) Pathway (KEGG, REACTOME and PANTHER) analysis of cancer associated genes (n=147) selected based on FC>2 and <-2 (FDR<0.1), identified using EnrichR (<http://amp.pharm.mssm.edu/Enrichr/index.html>).

**Supplementary Table S9:** Sequences of primers used for quantitative PCR analysis.
