# Supplementary material for: Transcriptome profiling of caspase-2 deficient EμMyc and Th-MYCN mouse tumors identifies distinct putative roles for caspase-2 in neuronal differentiation and immune signaling
Source: Cell Death Dis. 2019 Jan 22;10(2):56. doi: 10.1038/s41419-018-1296-0 (PMC6343006; doi:10.1038/s41419-018-1296-0)
Supplement: Supplementary file 2 — Supplementary figures [file 41419_2018_1296_MOESM2_ESM.pdf]

a

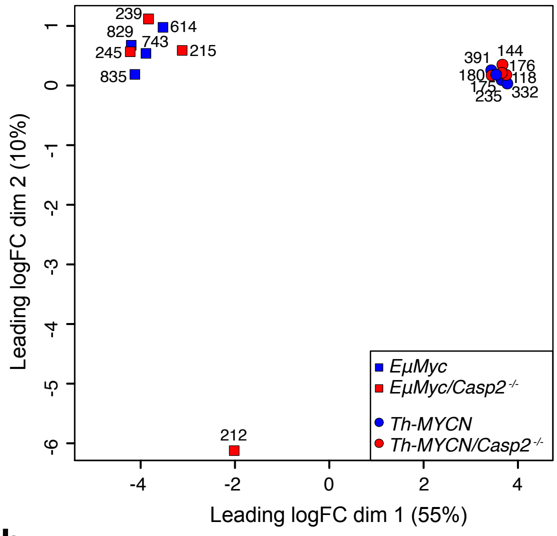

b

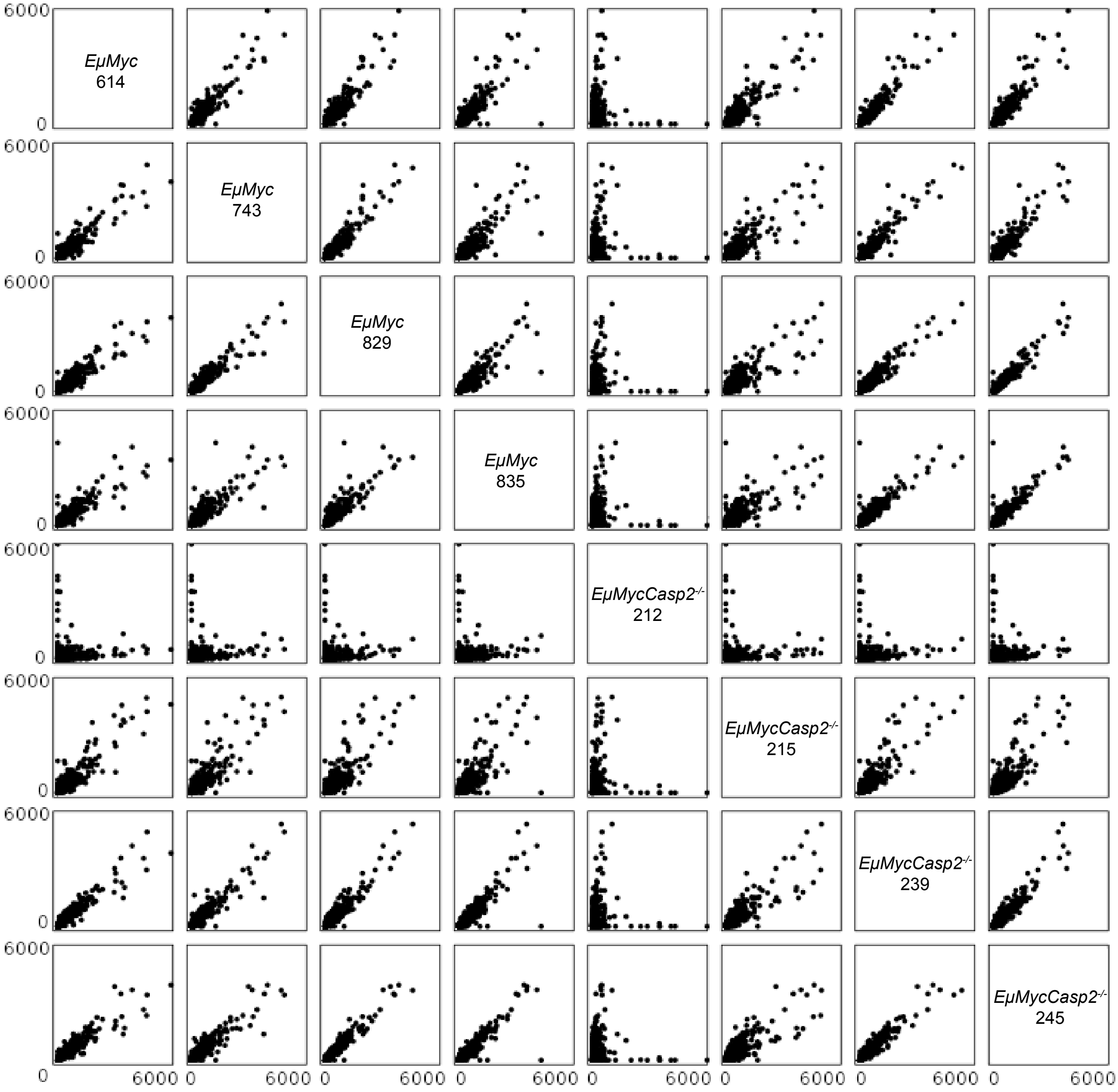

Supplementary Figure S1

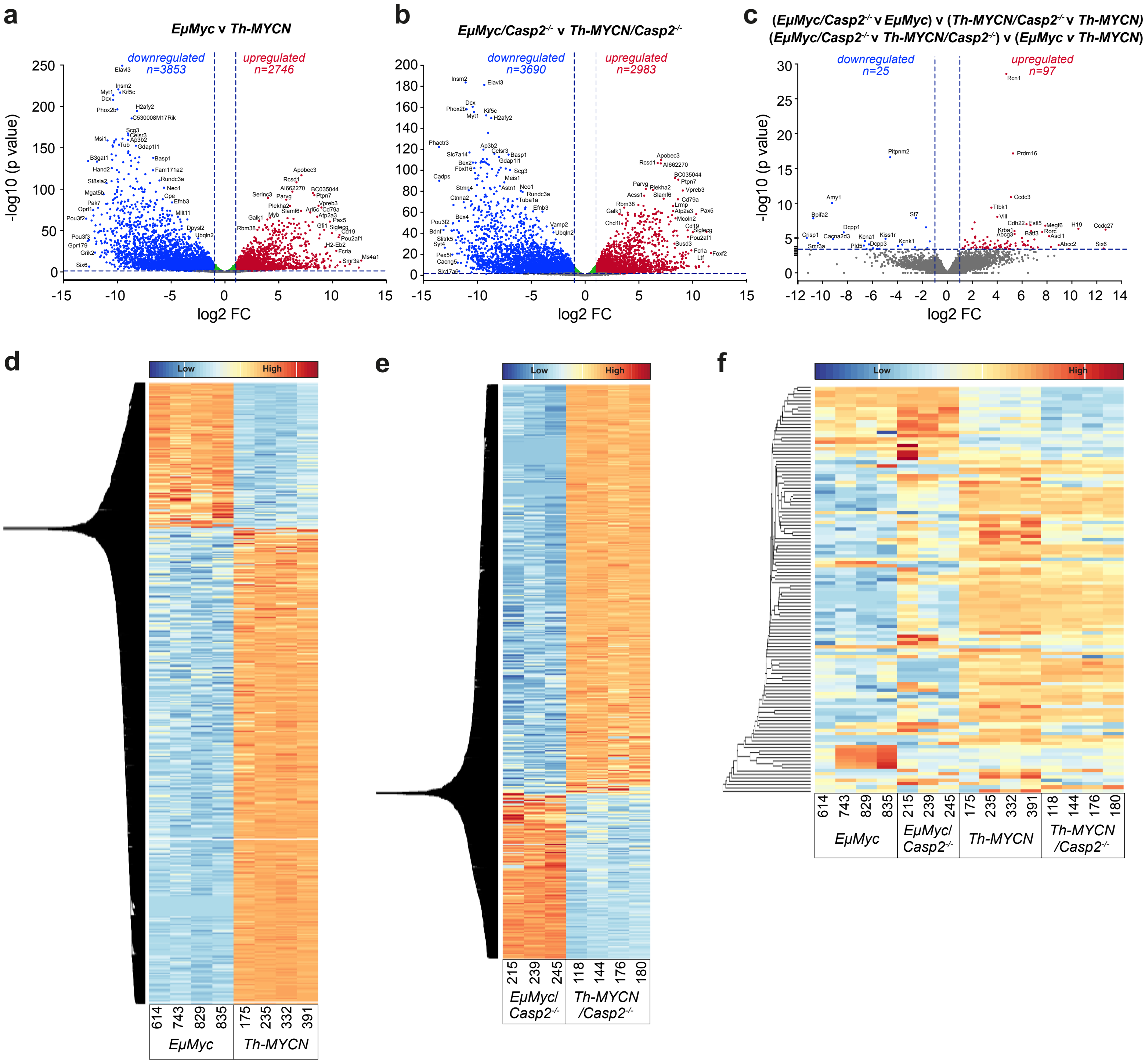

Supplementary Figure S2

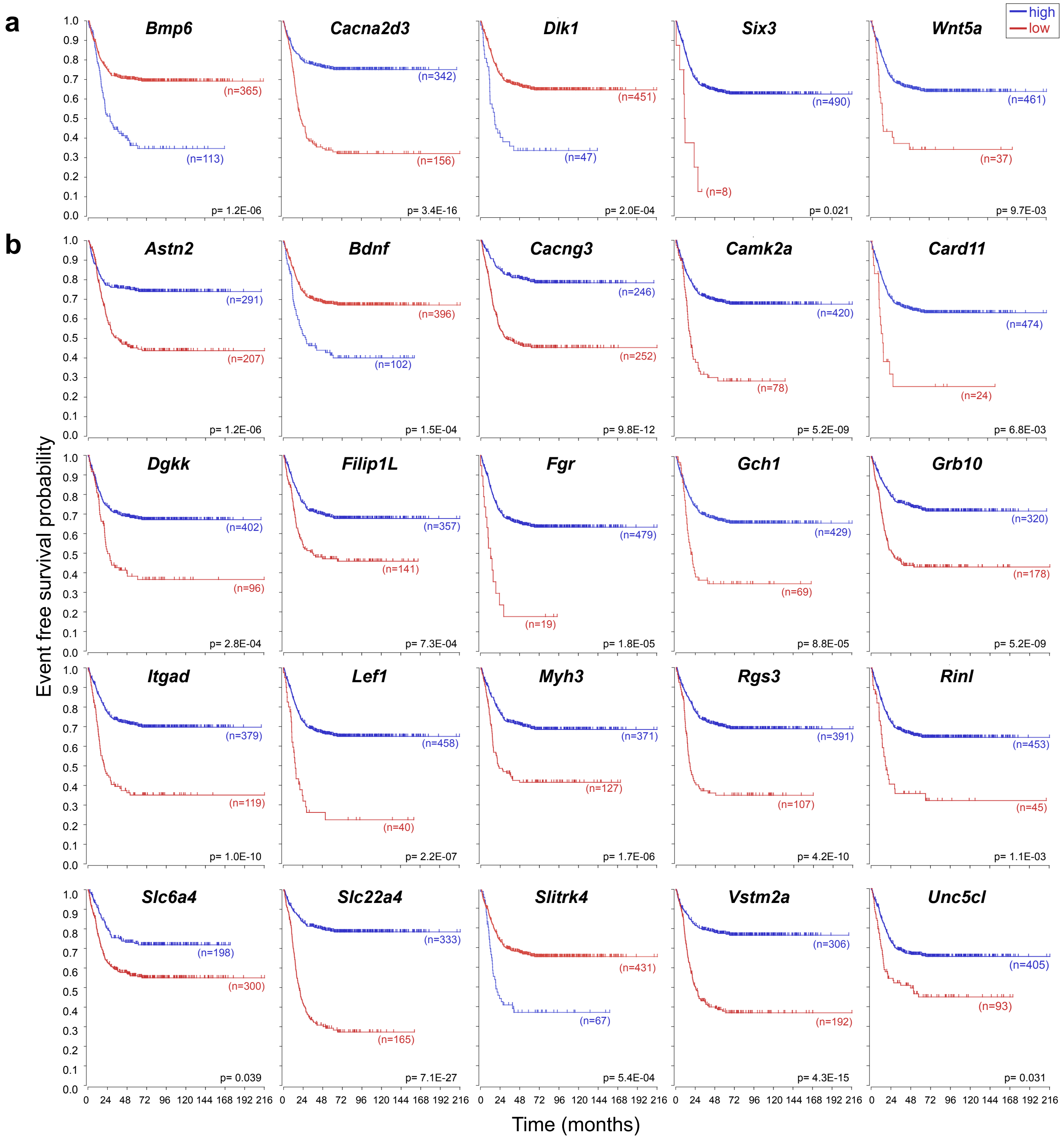

Supplementary Figure S3

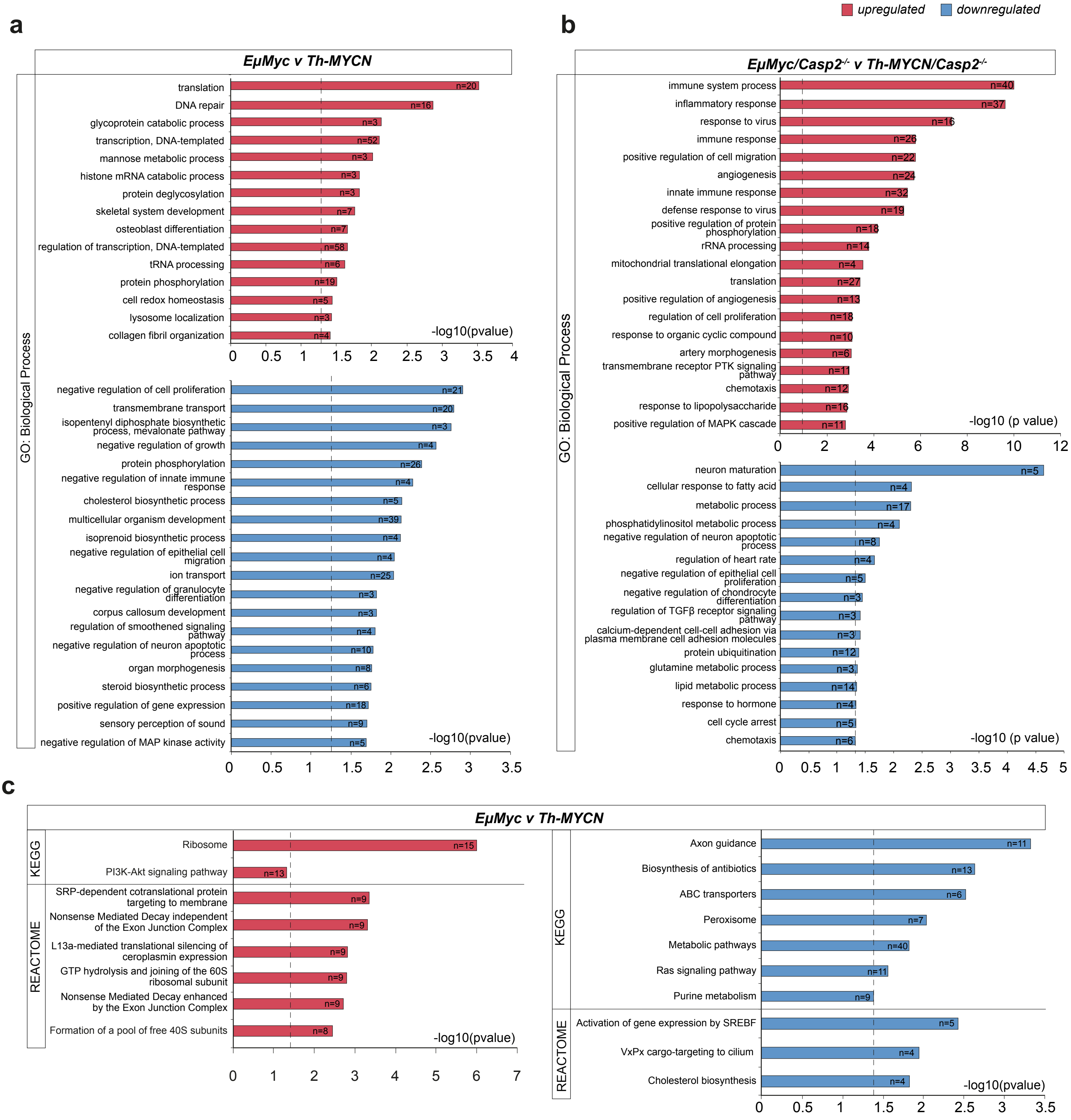

Supplementary Figure S4
